# Gap Junction in the Teleost Fish Lineage: Duplicated Connexins May Contribute to Skin Pattern Formation and Body Shape Determination

**DOI:** 10.3389/fcell.2017.00013

**Published:** 2017-02-21

**Authors:** Masakatsu Watanabe

**Affiliations:** Graduate School of Frontier Biosciences, Osaka UniversitySuita, Japan

**Keywords:** connexin, gap junction, skin pattern, bone shape, zebrafish

## Abstract

Gap junctions are intercellular channels that allow passage of ions and small molecules between adjacent cells. Gap junctions in vertebrates are composed of connexons, which are an assembly of six proteins, connexins. Docking of two connexons on the opposite cell surfaces forms a gap junction between the cytoplasm of two neighboring cells. Connexins compose a family of structurally related four-pass transmembrane proteins. In mammals, there are ~20 connexins, each of which contributes to unique permeability of gap junctions, and mutations of some connexin-encoding genes are associated with human diseases. Zebrafish has been predicted to contain 39 connexin-encoding genes; the high number can be attributed to gene duplication during fish evolution, which resulted in diversified functions of gap junctions in teleosts. The determination of body shapes and skin patterns in animal species is an intriguing question. Mathematical models suggest principle mechanisms explaining the diversification of animal morphology. Recent studies have revealed the involvement of gap junctions in fish morphological diversity, including skin pattern formation and body shape determination. This review focuses on connexins in teleosts, which are integrated in the mathematical models explaining morphological diversity of animal skin patterns and body shapes.

## Introduction

**Figure 1:**
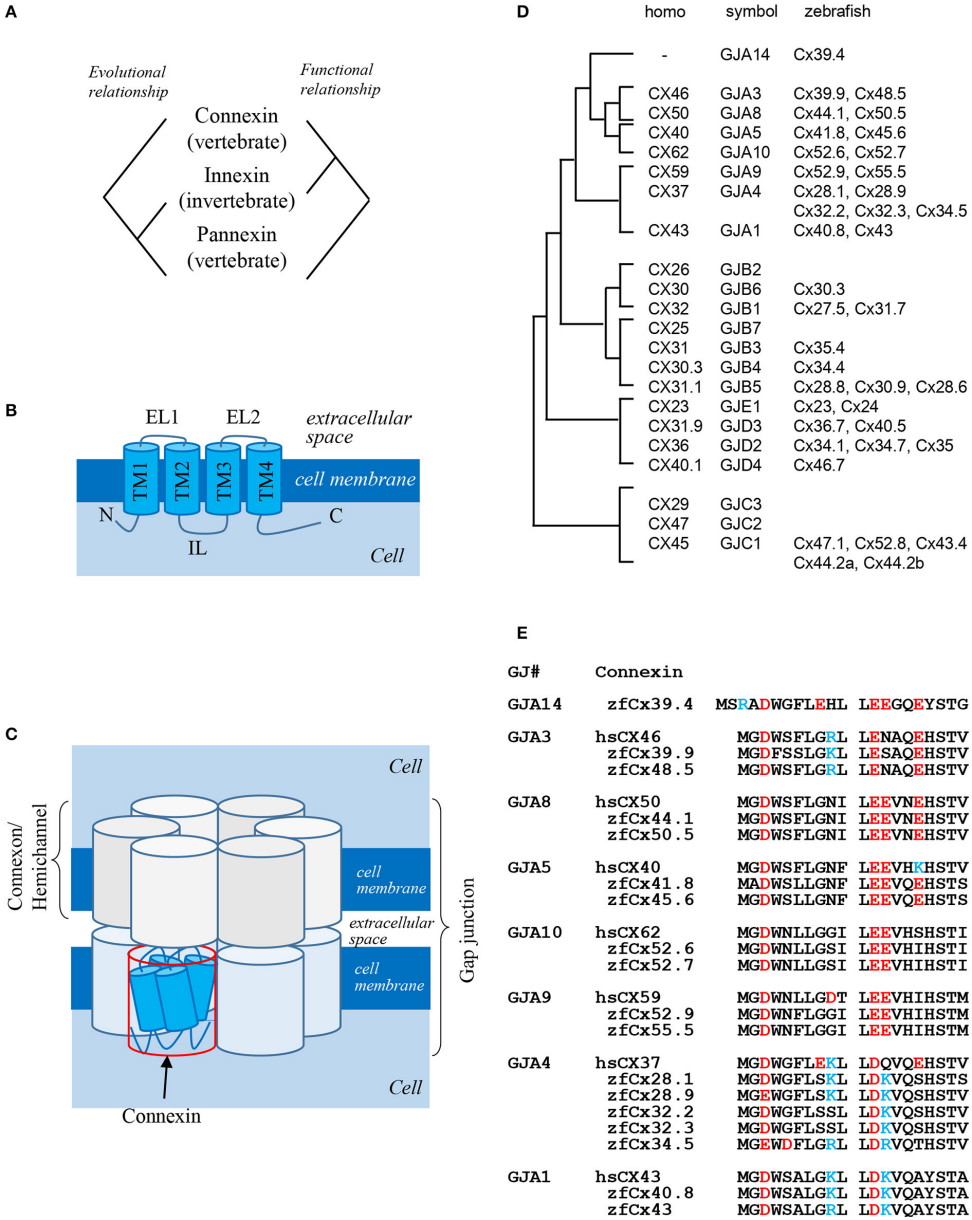
**Gap junction proteins. (A)** Relationship among three types of gap junction proteins: connexins, innexins, and pannexins (Baranova et al., 2004). **(B)** Structure of connexin proteins. N: N-terminus; TM: transmembrane domain; EL: extracellular loop; IL: intracellular loop; C: C-terminus (Kumar and Gilula, 1996). **(C)** Schematic presentation of a gap junction (Kumar and Gilula, 1996). **(D)** Phylogenic relationship between human and zebrafish connexins (Eastman et al., 2006; Cruciani and Mikalsen, 2007). **(E)** Sequence alignment of N-terminal domains of human and zebrafish alpha-type connexins (Connexin sequences were obtained from genome data base in Sanger Institute, http://www.sanger.ac.uk/).

Gap junctions are intercellular channels that mediate the transfer of small molecules between adjacent cells (Kumar and Gilula, 1996). Because of low size selectivity of molecules transferred through gap junctions (<1,000 Da), it is difficult to determine the biological functions of gap junctions in the organisms. Gap junctions are composed of two hemichannels formed by four-pass transmembrane proteins: connexins and innexins (Figures 1A,B; Baranova et al., 2004). Connexins are vertebrate-specific gap junction proteins, whereas innexins are expressed in invertebrates. Connexins as well as innexins form both hemichannels and gap junctions, while pannexins expressed in vertebrates but homologous to invertebrate innexins predominantly exist as hemichannels connecting the intracellular and extracellular space, rather than gap junctions connecting adjacent cells. Curiously, there is no evolutional relationship between connexins and pannexins, although both are expressed in vertebrates. In summary, connexins and innexins are functional homologs, while innexins and pannexins are evolutionary homologs (Figure 1A; Scemes et al., 2007).

Six connexin proteins form a hexamer called connexon, which functions as a hemichannel. After docking of two connexons on neighboring cell membranes, a gap junction is formed (Figure 1C). Connexins consist of several structural domains: the N-terminus, transmembrane region, extracellular and intracellular loops, and C-terminus (Figure 1B; Maeda et al., 2009). The N-terminal domain functions as a plug providing closure of gap junctions (Oshima et al., 2007) and as a voltage sensor of membrane potential (Verselis et al., 1994). The C-terminal domain has several phosphorylation sites which transmit signals to control the opening and closing of gap junctions and are also implicated in other biological pathways (Hebert and Stains, 2013), while extracellular loops are responsible for docking of hemichannels (Kumar and Gilula, 1996).

The number of connexin genes differs depending on animal species. In the human genome, there are 21 connexin genes, whereas in the zebrafish genome, 39 connexin genes are predicted (Hebert and Stains, 2013). Connexin proteins are named according to their molecular weight; for example, connexin 43 (Cx43) is a 43-kDa protein. This system sometimes causes confusion because orthologous genes belonging to different species have different gene symbols; thus, human *CX46* and zebrafish *cx39.9* are orthologous genes (Figure 1D). There is also other classification system based on gap junctions which are divided into five families, from gap junction alpha (GJA) to gap junction epsilon (GJE), and all connexins are named according to a specific subfamily (Figure 1D, symbol). In this system, orthologous genes in different animal species belong to the same gap junction subfamily. Although this classification makes it easier to understand the relationship between connexins and gap junctions, it has faults, because some connexins have a potential to form gap junctions with different connexin proteins. Thus, gap junctions composed of the same hemichannels consisting of two or more connexins are called heteromeric, while those composed of different hemichannels are called heterotypic. Although the formation of heteromeric-heterotypic gap junctions has been extensively examined in *in vitro* experiments, its role *in vivo* remains largely unknown.

## Connexins in teleosts

**Figure 2:**
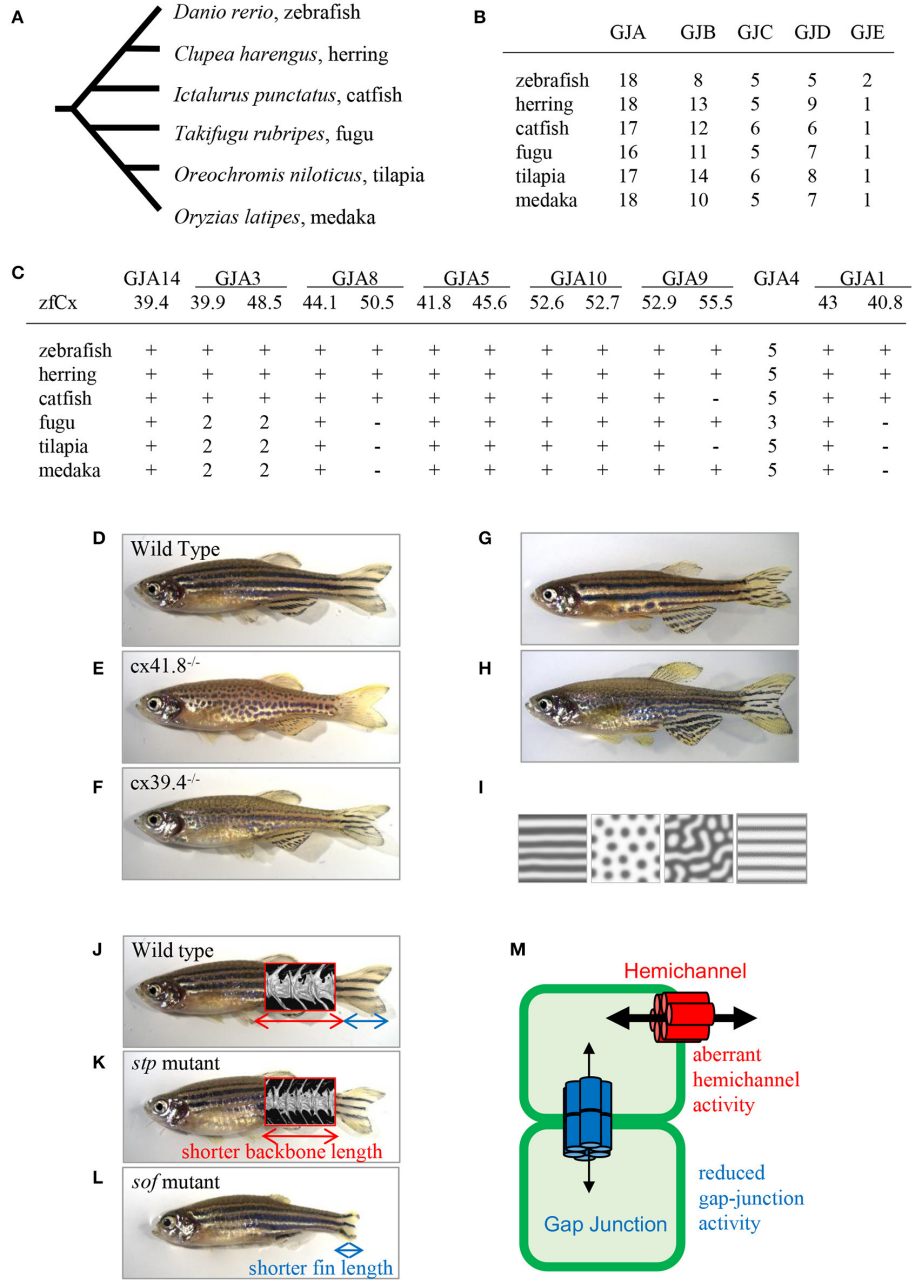
**Connexins in teleosts. (A)** Phylogenic relationship among six teleost species (Chen et al., 2004). **(B)** The number of connexin-encoding genes in six teleost species. **(C)** The number of genes encoding connexins of the alpha family gap junctions. “zfCx” indicates zebrafish connexins; “+” and “−” indicate the existence or absence, respectively, of an ortholog. If more than one orthologous gene was found, gene numbers are indicated (**B,C**; Connexin sequences were obtained from genome data base in Sanger Institute, http://www.sanger.ac.uk/). **(D–I)** Connexins in zebrafish pigment patterns. Wild-type zebrafish **(D**; Watanabe and Kondo, 2012); *leopard* mutant (**E**; Watanabe and Kondo, 2012); *luchs* mutant (**F**; Irion et al., 2014; Watanabe et al., 2016); transgenic zebrafish Tg(mitfa-cx41.8) >> *leopard* (**G**; Watanabe and Kondo, 2012); transgenic zebrafish Tg(mitfa-cx41.8M7) >> wild-type (**H**; Watanabe and Kondo, 2012); reaction-diffusion (R-D) patterns (**I**; Watanabe and Kondo, 2012). **(J–M)** Connexins in zebrafish bones; micro-CT images of vertebrae are superimposed. Wild-type zebrafish (**J**; Misu et al., 2016), *stp* mutant (**K**; Misu et al., 2016), *sof* mutant (**L**; Iovine et al., 2005; Misu et al., 2016). Schematic presentation of gap junction and hemichannel functions in zebrafish mutants (**M**; Misu et al., 2016). Red font, functional activity of hemichannels in the *stp*-Cx43 mutant; blue font, functional activity of gap junctions in the *sof*-Cx43 mutant.

There are more connexin-encoding genes in zebrafish than in humans (39 and 21, respectively; Eastman et al., 2006; Cruciani and Mikalsen, 2007). Here, I analyzed the number of connexin genes in six teleost species: zebrafish, herring, catfish, fugu, tilapia, and medaka using Genome Database (Ensembl, Sanger Institute). Zebrafish, herring, and catfish form one sister group, and the other three species form another one (Figure 2A). The results show that ~40 connexin genes exist in the genomes of the examined teleost species, although it is not known whether all the genes are expressed and functional. The duplication of connexin-encoding genes may have occurred in the ancestor of the teleost lineage through chromosome duplication events. After counting the number of connexin genes and categorizing them into the GJA–GJE subfamilies, it appeared that the examined teleost species had similar gene numbers in each subfamily (Figure 2B). Figure 2C shows the number of connexin genes belonging to the GJA subfamily. For example, *cx39.4* belongs to the GJA14 subfamily and all six species examined have one *cx39.4* ortholog in their genome. In the fugu-tilapia-medaka lineage, connexins of the GJA3 subfamily were duplicated and *cx40.8* and *cx50.5* orthologs were lost in their common ancestor. On the other hand, *cx55.5* might have been lost in catfish and tilapia independently. Connexins belonging to the GJA4 subfamily have three or more paralogous genes. The high copy numbers of GJA4 paralogous genes may be explained by local chromosomal duplications occurred in the ancestor. In addition to the high copy number, the *cx39.4* gene is teleost-specific and is not detected in human, chicken, lizard, or *Xenopus* genomes (Figures 1D,E).

## Connexins in zebrafish

Zebrafish is a small tropical fish with a body 3–4-cm long. Because of its transparent embryo, easy breeding, generation of transgenic lines, and availability of genomic resources through advances in genome sequencing technology, zebrafish is considered an important and convenient model organism for developmental studies in vertebrates. However, knocking out of some connexin gene shows no phenotypes in zebrafish, probably because of gene redundancy or other reasons. To date, the expression of several connexin genes was identified in zebrafish mutants: *cx39.9* in muscle (Hirata et al., 2012), *cx52.6* and *cx55.5* in retina (Klaassen et al., 2011), *cx43* in fins and vertebrae (Iovine et al., 2005; Misu et al., 2016), and *cx36.7* in the heart (Sultana et al., 2008), while *cx41.8* and *cx39.4* were found to be responsible for pigment pattern (Watanabe et al., 2006; Irion et al., 2014).

## Connexins in zebrafish pigment pattern

One of the famous characteristics of zebrafish is “zebra” stripe observed on the skin surface (Figure 2D), and the mechanism underlying the generation of the stripe pattern has long been an intriguing question. Sixty years ago, English mathematician Alan M. Turing proposed a mathematical model called the reaction-diffusion (R-D) model, which explained the mechanism underlying pattern formation (Turing, 1952) and which was later applied to biological phenomena (Kondo and Asai, 1995). This mathematical model represents the interaction and diffusion of two hypothetical factors, allowing, by changing the parameters in the equations, to generate various patterns *in silico*. Zebrafish stripes are made of two types of pigment cells, melanophores and xanthophores, and it is shown that interactions between these pigment cells satisfy the condition of the R-D model (Yamaguchi et al., 2007; Nakamasu et al., 2009). Thus, it can be assumed that the zebrafish skin pattern is generated in the R-D manner, which makes zebrafish a model organism for pattern formation studies.

One of the most famous zebrafish skin pattern mutants is *leopard* fish, which has spots instead of stripes (Figure 2E). This mutant was originally identified from field and several alleles were isolated from mutagenesis pools (Haffter et al., 1996). Because the spot is a representative pattern of the R-D model (Figure 2I), the *leopard* mutant is an important target of pattern formation studies (Asai et al., 1999). Ten years ago, our group identified the gene responsible for the *leopard* pattern, which encoded a gap junction protein Cx41.8 (Watanabe et al., 2006), a zebrafish ortholog of mammalian CX40 and a paralog of zebrafish Cx45.6 (Eastman et al., 2006). Knocking out *cx41.8* results in a spotted skin pattern, whereas knocking out *cx45.6* does not produce a skin phenotype. It should be noted that molecules functioning in cell–cell interaction should participate in stripe-to-spot changes *in vivo* because such changes are predicted by the mathematical model for the interaction between two hypothetical factors. This notion was confirmed when we successfully generated the R-D patterns on zebrafish skin using Cx41.8 mutants (Figures 2G–I; Watanabe and Kondo, 2012). To further investigate the role of gap junctions in skin pattern formation, we constructed transgenic fish lines in which connexin-encoding genes were ectopically expressed in pigment cells of the *leopard* fish (Watanabe et al., 2012). The results indicated that, in addition to *cx41.8*, other genes such as *cx44.1, cx45.6*, and *cx48.5* rescued the *leopard* phenotype, while *cx27.5, cx30.8, cx32.2*, or *cx43* did not. Amino acid alignment of N-terminal connexin domains revealed that connexins which rescued the *leopard* phenotype belonged to the GJA14, GJA3, GJA8, and GJA5 subfamilies as evidenced by the presence of the ExxxE motif (Figure 1E), a polyamine-binding site important for rectifying properties of gap junctions (Musa et al., 2004).

Polyamines, mainly putrescine, spermidine, and spermine, are small molecules important for cell proliferation and differentiation; they are known to regulate K+ inward-rectifier (Kir) channels through binding to the channel pore (Hibino et al., 2010). We have isolated Kir7.1 from a zebrafish skin pattern mutant, *jaguar*, and shown that the Kir7.1 channel is expressed in melanophores, where it forms resting potential (Iwashita et al., 2006); this hyperpolarization is important for the generation of a clear boundary between melanophores and xanthophores on fish skin (Inaba et al., 2012). Based on this finding, we introduced the *ssat* gene encoding polyamine metabolic enzyme spermidine/spermine N1-acetyltransferaseinto melanophores, and found that the ectopic expression of *ssat* disturbed the stripe pattern of zebrafish (Watanabe et al., 2012). Interestingly, *ssat*-expressing transgenic zebrafish showed a unique phenotype of large spots and wide stripes, which is an intermediate pattern between the *leopard* (*cx41.8*) and *jaguar* (*kir7.1*) mutants (Iwashita et al., 2006; Watanabe et al., 2006, 2012). Furthermore, a recent study showed that spermidine synthase was also involved in skin pattern formation as confirmed by the isolation of spermidine, but not spermine, from melanophores (Frohnhofer et al., 2016). Taken together, these data indicate that spermidine may bind both Kir7.1 and Cx41.8, and control the rectification properties of Kir7.1 as well as of Cx41.8 gap junctions in melanophores. As a result, the expected unidirectional functioning of gap junctions from xanthophores to melanophores would be provided, which is consistent with a previous observation that xanthophores are required for melanophore survival (Nakamasu et al., 2009).

Cx39.4 is another connexin protein shown to be involved in the skin pattern formation of zebrafish. Cx39.4 is the teleost lineage-specific connexin (Figures 1D,E) recently isolated from a zebrafish skin pattern mutant, *luchs* (Figure 2F) (Irion et al., 2014). We examined the expression of alpha-type connexins in pigment cells and found that the *cx41.8* and *cx39.4* genes were expressed in melanophores and xanthophores, which was also confirmed in transgenic zebrafish carrying a reporter gene under connexin promoters. To compare Cx39.4 and Cx41.8 functions in skin pattern formation, we performed complementation experiments when *cx39.4* was introduced into the *leopard* mutant and *cx41.8* into the *luchs* mutant (Watanabe et al., 2016). None of them was able to rescue each other phenotypes, indicating that Cx41.8 and Cx39.4 have distinct functional activities. Although Cx39.4 contains the N-terminal ExxxE motif important for pattern formation, the sequence of its N-terminus is unique because it is two residues longer than that in other alpha-type connexins and has a basic residue at the third position. Our electrophysiological analysis showed that large voltage-dependent current was absent in Cx39.4-expressing oocytes, indicating that the basic residue at the third position affected the characteristics of gap junctions and accounted for the difference of channeling properties between Cx39.4 and Cx41.8 (Watanabe et al., 2016).

## Connexins in zebrafish bones, fins, and vertebrae

The variation in body shape among animal species has long been an intriguing question. A century ago, Scottish mathematician and biologist D'Arcy Thompson proposed the theory of transformations suggesting that new body shapes arise by changing angles, extending the length, or enlarging body parts, pointing out correlations between biological forms and mechanical phenomena (Thompson, 1961). Bones determine body shape in vertebrates, and zebrafish fins present a valuable model for the study of bone organogenesis and regeneration because of their rapid growth. Recent advances in transgenic techniques enable the detection of gene expression in bone-producing cells during bone growth and regeneration in live fish. Among the connexin family members expressed in zebrafish, Cx43 is known to be involved in the formation and regeneration of the fin and in determining vertebra proportions (Iovine et al., 2005; Misu et al., 2016).

Ten years ago, zebrafish Cx43 was identified in the *short-of-fin* (*sof*) mutant who has a short fin segment (Figure 2L; Iovine et al., 2005). Four *sof* alleles were isolated from zebrafish mutagenesis pools and amino acid substitutions causing the loss or decrease of Cx43 gap junction functional activity were identified in three mutant alleles. On the other hand, no mutation was found in the coding region of *cx43* in the fourth allele; however, the downregulation of both mRNA and protein expression were detected in the carriers of this allele. Overall, these findings indicate that a decrease of Cx43 gap junction properties produces the short-fin phenotype (Figure 2L). In addition, Cx40.8, a paralog of Cx43 in zebrafish is also involved in fin development and regeneration (Gerhart et al., 2009). Interestingly, although the biological function of Cx40.8 is very similar to that of Cx43, its membrane localization is differentially controlled depending on the developmental phases and regeneration status (Gerhart et al., 2012).

To provide deeper understanding of the molecular mechanisms supporting the theory of transformations, our group focused on a zebrafish body-shape mutant named *stoepsel* (*stp*; Figure 2K) identified 20 years ago from a zebrafish mutagenesis pool based on reduced body length. Recently, we performed precise analysis of bone shape development in this mutant fish. Micro-CT scanning images revealed that the vertebra shape of the *stp* mutant was almost the same as that of the wild-type fish, although the vertebra size along the anterior-posterior (A-P) axis was decreased (Figures 2J,K). Because the vertebra height along the dorsal-ventral (D-V) axis was unchanged, this fish presents a proportion mutant according to the theory of transformations. We also found that the mutant phenotype appeared 50 days post-fertilization, indicating that it is expressed in the adulthood. To disclose the underlying molecular mechanism, we performed positional cloning experiments and identified a point mutation in the *cx43* coding region. Then, we asked a question why mutations occurring in same *cx43* gene caused different phenotypes, i.e., short fin or short vertebra. To address this question, we compared the functions of gap junctions and hemichannels by performing dual-cell voltage clamp experiments and found that the functional activity of *stp*-Cx43 gap junction was decreased similar to that of the *sof*-Cx43 mutant (Misu et al., 2016). Measurements of fin segment length in the *stp* mutant revealed that it was 5% shorter than that of the wild-type fish (Misu et al., 2016), which is consistent with previous findings that shortening of the fin segment is proportional to the reduction in gap junction function (Hoptak-Solga et al., 2007). On the other hand, we detected aberrant increase of *stp*-Cx43 hemichannel activity, while no difference was detected between *sof*-Cx43 and the wild-type Cx43. These findings suggest that malfunctioning of hemichannels results in reduced backbone length, whereas a decrease in gap junction activity causes shortening of the fin segment (Figure 2M), although the underlying mechanism remains unclear (Misu et al., 2016).

The mutation in the human *GJA1* gene encoding CX43 is known to be responsible for an extremely rare disease, oculodentodigital dysplasia (ODDD), manifested by small eyes, underdeveloped teeth, and malformation of fingers (Paznekas et al., 2003). As zebrafish Cx43 is an analog of human CX43, zebrafish can present a good experimental model to study ODDD and would help understand the mechanisms controlling bone formation.

## Mathematical models predict gap junction functions in pattern formation

It is very interesting that two independent projects which aimed to disclose the molecular mechanism hidden in mathematical models, found the involvement of gap junction proteins: Cx41.8 in the R-D model and Cx43 in the theory of transformation.

In the R-D model, positive interactions between two factors are important. As mentioned above, Cx41.8-gap junctions might be formed between xanthophores and melanophores, and control the directional flow of small molecules from a xanthophore to a melanophore depending on spermidine concentration. In addition, the R-D model predicts that Cx41.8 might be involved in melanophore differentiation. The reduction of basal level synthesis of a factor also causes pattern changes in the R-D model (Asai et al., 1999), and the reduction of the number of melanophores are observed in the *leopard* mutant (Figure 2E).

Regarding the theory of transformation, the *stp* mutant might be the simplest example. It is possible that the reduction of osteogenic activity causes the formation of shorter vertebra in the *stp* mutant, although the difference in the mechanisms underlying the formation of shorter vertebra in the *stp* fish and shorter fins in the *sof* fish is unclear. Recent gap junction studies have revealed that hemichannels formed by connexins and/or pannexins function as sensors of mechanical stress (Jiang et al., 2009; Thi et al., 2012), which supports the possibility that gap junctions control body shape variations, including shortening, expanding, and twisting of body frames as predicted by the theory of transformation.

Many questions still remain, including functional differences among connexin proteins, the type of transferred molecules, and the evolutionary events that have led to the acquisition of this intercellular communication system in the animal kingdom. Future mechanistic and genetic studies would address these questions.

## Author contributions

The author confirms being the sole contributor of this work and approved it for publication.

### Conflict of interest statement

The author declares that the research was conducted in the absence of any commercial or financial relationships that could be construed as a potential conflict of interest.
